# Development of High-Temperature Wire-Grid Thin Film Strain Gauges

**DOI:** 10.3390/s22197595

**Published:** 2022-10-07

**Authors:** Yunxian Cui, Xin Li, Tenglun Zhang, Wanyu Ding, Junwei Yin

**Affiliations:** 1 School of Mechanical Engineering, Dalian Jiaotong University, Dalian 116028, China; 2 Nanjing Kangni Mechanical & Electrical Co., Ltd., Nanjing 210013, China; 3 School of Materials Science and Engineering, Dalian Jiaotong University, Dalian 116028, China

**Keywords:** thin film, strain gauge, high-temperature, nanocomposite insulation coatings, magnetron sputtering

## Abstract

Aero-engine turbine stator blades are often used in harsh environments with high temperatures and high pressure and are prone to fatigue fractures. Real-time and accurate monitoring of blade surface stress and strain is critical to ensure safe operation. In this study, thin-film strain gauges (TFSGs) that can be used in high-temperature environments above 1000 °C were designed and fabricated using a PtRh6 thin film as the sensitive material. The hysteresis effect of the stress transfer upon establishing a thermo-mechanical coupling finite element model of the Inconel718 high-temperature nickel-based alloy equal-strength beam PtRh6 TFSGs was analyzed and the optimal combination of thin-film thickness and longitudinal grid length of wire-grid TFSGs was determined. In order to solve the problem of high-temperature insulation, the insulating properties of a single-layer Al_2_O_3_ insulating film, a single-layer ZrO_2_ insulating film, a double-layer Al_2_O_3_/ZrO_2_ composite insulating film, and a four-layer Al_2_O_3_/ZrO_2_/Al_2_O_3_/ZrO_2_ composite insulating film at high temperature were compared and studied using scanning electron microscopy to analyze the microscopic morphology and composition of the four insulating film structures. The results showed that the four-layer Al_2_O_3_/ZrO_2_/Al_2_O_3_/ZrO_2_ composite insulating film had the best insulating properties at high temperatures. On this basis, an Al_2_O_3_/ZrO_2_/Al_2_O_3_/ZrO_2_ composite insulating film, PtRh6 sensitive layer, and Al_2_O_3_ protective film were sequentially deposited on a high-temperature nickel-based alloy equal-strength beam using DC pulsed magnetron sputtering technology to obtain an Inconel718 high-temperature nickel-based alloy equal-strength beam PtRh6 TFSG. Its gauge factor (GF) and temperature coefficient of resistance (TCR) were calibrated, and the results showed that the sensor could be used in harsh environments of 1000 °C. The above results provide new ideas for measuring stress and strain in aerospace under high-temperature and high-pressure environments.

## 1. Introduction

The working temperature of a turbine engine can exceed 1000 K and the pressure can exceed 40 bar [1]. The entire turbine stator blade bears the thermal stress, aerodynamic load, and vibration generated by rotating machinery, which directly affects the service life of the engine and the safety of the aircraft. Therefore, thin-film sensors for measuring a turbine engine’s surface strain, temperature, and heat flux were studied [2,3,4]. Conventional wire-type resistance strain gauges, berth-type resistance strain gauges, and fiber Bragg gratings (FBGs) [5] have a rough adhesive process, poor adhesion, inaccurate output signals, and cannot meet the strain measurement requirements for turbine static blades.

Thin-film strain gauges (TFSGs) can be deposited directly on a substrate surface using a magnetron sputtering technique to provide real-time accurate surface parameters due to their small size, low impact by airflow scouring, and strong adhesion [6]. Metals, semiconductors, and ceramics can be used as sensitive materials for thin-film strain gauges. However, so far, only a few high-temperature thin-film strain gauge materials have been reported, such as Ni80Cr20 [7,8], PdCr [9], TAN-Cu [10], and TiAlN [11], but the oxidation resistance is poor and the electrical performance decreases at high temperatures. Ni80Cr20 and TiAlN are used at temperatures below 600 °C. Pt [12,13] is used in the high-temperature environment of 500 °C–900 °C and is easy to oxidize and dissolve but is unsuitable for long-term use at high temperatures. ITO [14,15,16] has high stability and excellent resistivity, but its own electrical and chemical properties fluctuate considerably at temperatures below 950 °C with resistance drift, which affects the accuracy of the strain output results. Typical silicon-based/semiconductor strain gauges [17] have a higher GF and higher resistivity. The high values make it difficult to match deformable components and the components are susceptible to manufacturing tolerances, which reduces the overall sensitivity and accuracy. To solve the high-temperature problem, polymer-derived ceramics (PDCs) [18,19,20] also captured the attention of researchers with their tunable electrical properties and microstructures. However, the considerable volume shrinkage (40%–70%) during the pyrolysis of PDCs makes manufacturing thin films of PDCs a significant challenge [21].

The alloy PtRh6 has a low-temperature coefficient of resistance, high resistivity, and a high melting point. When the content of Rh in the alloy is less than 25%, it has stable high-temperature mechanical properties, can inhibit the volatilization of Pt under a high-temperature environment, and increases the thermoelectric potential of Pt. This alloy may better meet the needs of thin-film strain gauges at high temperatures, but PtRh6 TFSGs have rarely been reported.

In this study, a PtRh6 TFSG simulation model was established, transfer hysteresis analysis was performed, and the optimal strain gauge structure was determined. Through thermo-mechanical coupling, the stress variation trend, TCR, and GF at different temperatures were derived. Based on this, four layers were prepared on an Inconel718 nickel-based high-temperature alloy substrate using magnetron sputtering technology to form an Al_2_O_3_/ZrO_2_/Al_2_O_3_/ZrO_2_ composite insulating film for a PtRh6 high-temperature TFSG. For the prepared Al_2_O_3_/ZrO_2_/Al_2_O_3_/ZrO_2_ composite, the insulation layer structure, surface morphology, composition content, and high-temperature insulation characteristics were analyzed; the effects of heat treatment on the surface morphology and high-temperature insulation characteristics were compared; and the TCR and GF were evaluated.

## 2. Simulation Analysis and Structural Design

### 2.1. Material Selection

**Figure 1:**
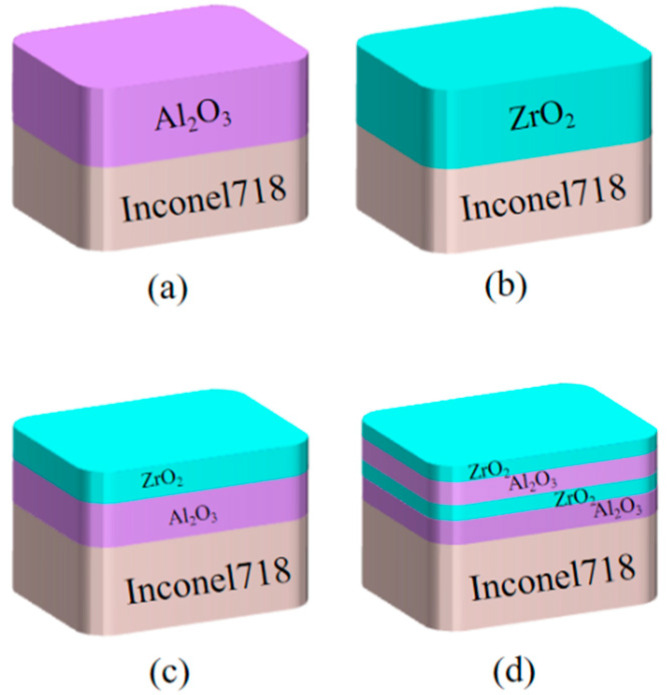
Schematic diagram of the insulating film structure: (**a**) single-layer Al_2_O_3_, (**b**) single-layer ZrO_2_, (**c**) double-layer Al_2_O_3_/ZrO_2_, and (**d**) four-layer Al_2_O_3_/ZrO_2_/Al_2_O_3_/ZrO_2_.

In this study, an Inconel 718 high-temperature nickel-based alloy with Young’s modulus of 227.79 GPa, Poisson’s ratio of 0.3241, and a density of 8.29 g/cm^3^ was selected as the substrate for the preparation of thin-film strain gauges cut into 70 mm × 10 mm × 3 mm equivalent strength beam structures. Al_2_O_3_ [22,23], which has good corrosion resistance and high chemical stability, was used as the insulating layer material to prevent the sensor from conducting with the substrate and affecting the measurement accuracy. However, single-layer insulating films are prone to voids or gaps [24,25], and the sensitive layer and the alloy substrate are connected. Therefore, a ZrO_2_ material with a high melting point and resistivity was selected to prepare a multilayer insulating film, and the grain complementation between different film materials was used to fill and repair the penetration defects in the film. With its compactness [26,27,28], the film achieved the purpose of high-temperature insulation. Four types of insulating layer films were prepared: a single-layer Al_2_O_3_ film, single-layer ZrO_2_ film, double-layer Al_2_O_3_/ZrO_2_ composite insulating film, and four-layer Al_2_O_3_/ZrO_2_/Al_2_O_3_/ZrO_2_ composite insulating film. Figure 1 shows the structure schematic diagram of the insulating layer structures. To improve the accuracy of the measurement results, avoid measurement errors caused by oxidation and other factors, and prolong the service life of the strain gauge, Al_2_O_3_ was used as a protective layer for the strain gauge.

### 2.2. Determination of the Simulation Boundary Conditions

**Figure 2:**
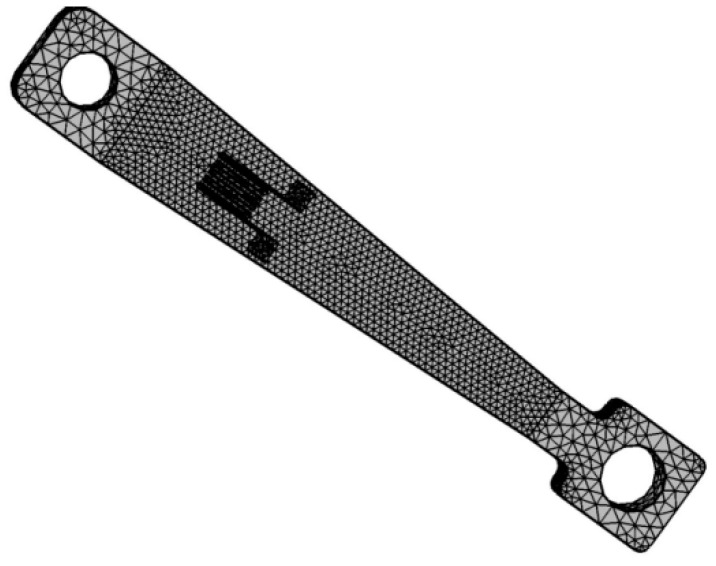
Equal-strength beam PtRh6 TFSGs finite element geometry model meshing.

We imported the Solidworks 3D assembly model into the simulation software and used the “solid mechanics” and “solid heat transfer” interfaces for the thermo-mechanical coupling analysis. Because the strain gauge was only a few microns thick, we used the “membrane” interface and the thin thermal approximation. The solid mechanics interface simulated the microstrain generated by the strain gauge after the load was applied. The solid heat transfer interface simulated the high-temperature environment in which the strain gauge was located. The ambient temperature of the strain gauge was set to 25 °C, 200 °C, 400 °C, 600 °C, 800 °C, and 1000 °C; we set the wide end of the equal-strength beam as a fixed constraint and applied a force per unit area at the other end; and we calculated the resistance change by calculating the deformation of the strain gauge. The material parameters were added in the simulation software’s “Material Library”. Figure 2 shows the finite element geometric and mesh division models of the equal-strength beam PtRh6 TFSGs. The equations used for the finite element model were as follows:

1. Heat conduction equation:
  (1) $${\rho c}_{p}u\cdot \nabla T=\nabla \cdot \left(k\cdot \nabla T\right)+Q$$
  where ρ is the mass density, $c_{p}$ is the specific pressure heat capacity, Q is the heat flow rate, u is the particle flows velocity, k is the thermal conductivity, and T is the solid surface temperature.
2. Heat flow conduction equation:
  (2) $$-n\cdot \left(-k\nabla T\right)=h\left(T_{\mathrm{ext}}-T\right)$$
  where n is the direction vector, h is the heat transfer coefficient, and $T_{\mathrm{ext}}$ is the air field temperature.
3. Thermal strain equation:
  (3) $$\varepsilon_{\mathrm{th}}=\alpha \left(T\right)\left(T-T_{\mathrm{ref}}\right)$$
  where $\varepsilon_{\mathrm{th}}$ is the thermal strain, α(T) is the coefficient of thermal expansion, and $T_{\mathrm{ref}}$ is the volume reference temperature.
4. Equation for calculating the amount of change in resistance:
  (4) $$R=\rho \frac{L}{\mathrm{hw}}$$
  where ρ is the resistivity, L is the gate length, h is the film thickness, and w is the gate width.

### 2.3. Simulation of the Transfer Hysteresis Effect

Due to the thickness difference between the substrate surface and the strain gauge, when the substrate surface is subjected to force and strain occurs, the strain output value cannot be fully transmitted to the strain gauge, which is a phenomenon known as the transfer hysteresis effect of TFSGs. The shear hysteresis effect [29,30] will reduce the test performance of the sensor itself. To accurately analyze the influence of the transmission lag effect on the strain transmission, all materials were set as linear elastic materials and isotropic materials; there was no relative sliding between the insulating layer, the sensitive layer, and the substrate; and the substrate is only stretched uniformly along the length of the sensitive gate.

**Figure 3:**
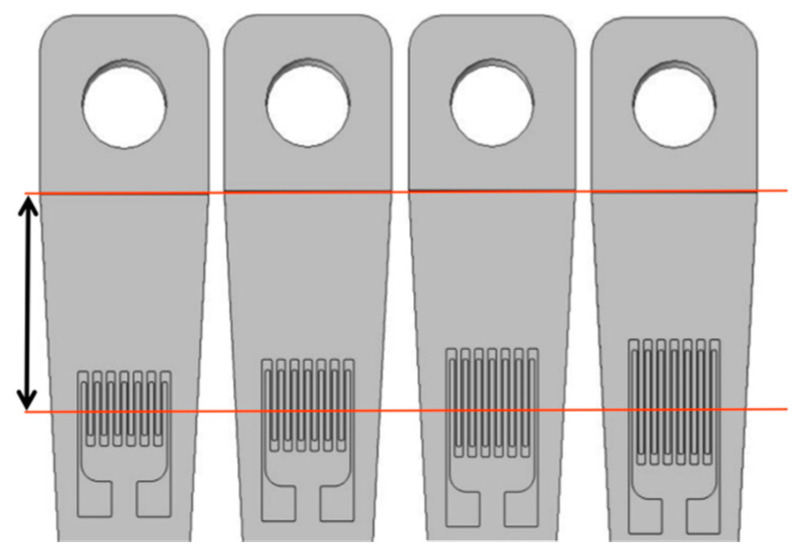
Model diagrams of different structure sizes.

Four different lengths and thicknesses of sensitive layer films were designed. A strain gauge with a total resistance of 120 Ω was created to match the XL2101B2 static strain gauge and to ensure that the widths of the transverse, electrode, and longitudinal grid zones were constant. Using Solidworks modeling and simulation analysis, the sizes of the sensitive layer functional areas were calculated (Table 1) using the boundary probe of the simulation software to monitor and compare the output strain value of the substrate surface. To avoid inaccurate strain output results due to design errors in the substrate, the midlines of the longitudinal grids of the four sensitive layers were made sure to coincide with the same intercept line of the substrate (Figure 3). Table 2 shows the physical parameters of each functional layer material of the TFSG.

**Figure 4:**
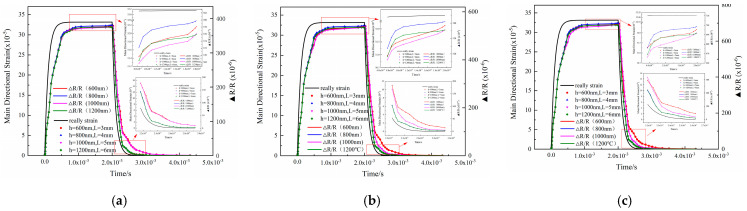
Comparison of the strain transfer hysteresis errors at different temperatures: (**a**) 200 °C, (**b**) 600 °C, and (**c**) 1000 °C.

In order to obtain a complete loading and unloading strain pulse signal, a force of 25 N was applied to the substrate. Figure 4 depicts the hysteresis error of the strain transfer at different temperatures with different film thicknesses and gate lengths. There were errors in the loading and unloading process due to the lag of the strain transmission. When loading, the simulated strain output of the sensitive layer with a thickness of 800 nm was almost identical to that of the substrate surface. In comparison, the strain error of the sensitive layer with a thickness of 1000 nm was relatively large. When unloaded, the shapes of the strain curves of the sensitive layers with thicknesses of 800 nm and 1200 nm were similar. When the unloading temperature exceeded 200 °C, the simulated strain output values were close to the actual strain output values. When the unloading reached a steady state, the strain transfer error of the sensitive layer with a thickness of 600 nm grew steadily with increasing temperature. The strain transfer error of the sensitive layer with a thickness of 600 nm was the largest.

**Figure 5:**
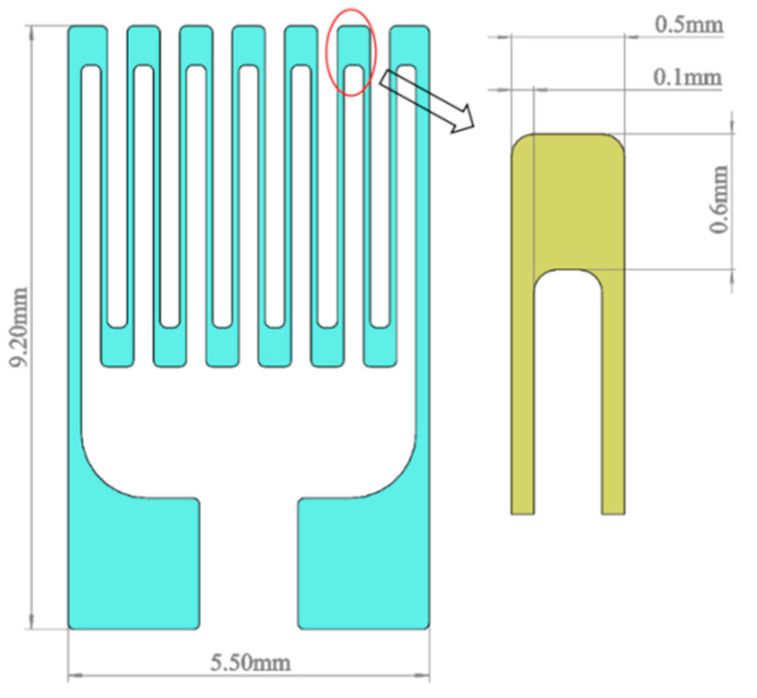
Structure diagram of the sensitive layer.

The steady-state strain outputs of sensitive layers with different film thicknesses at different temperatures are shown in Table 3. Once the load reached its steady-state value, the temperature had almost no effect. When the unloading reached a steady state, the strain errors of the sensitive layers with thicknesses of 600 nm, 1000 nm, and 1200 nm increased with temperature, while those of the sensitive layers with thicknesses of 800 nm were relatively small at all other temperatures except for 600 °C. Therefore, this study used a film thickness of 800 nm. Considering the above factors, the structure of a thin-film sensitive layer is depicted in Figure 5. Detailed parameters of each functional area are shown in Table 4.

### 2.4. Equal-Strength Beam Thermo-Mechanical Coupling Simulation of PtRh6 TFSGs

Thermo-mechanical coupling refers to the interaction between two physical fields, i.e., the stress field and the temperature field. In this study, the effect of temperature on the film stress was studied, the thermal expansion coefficients of the Inconel718 high-temperature nickel-based alloy and PtRh6 alloy at different temperatures were linearly fitted, and the fitted function was input into the simulation software. The linear thermal expansion coefficients of the two materials at temperatures of 100 °C–1000 °C are shown in Table 5 [31].

**Figure 6:**
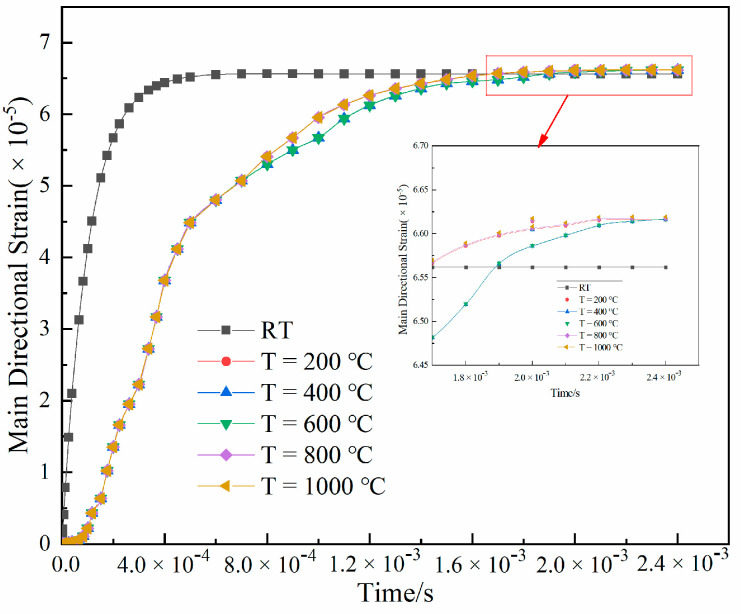
Equal-strength beam PtRh6 high-temperature thin film strain gauge transient strain curve.

A minimum force of 5 N was applied to the Inconel718 high-temperature nickel-based alloy equal-strength beam to better observe the variation law of the strain output at different temperatures. As shown in Figure 6, after 1.6 × 10^−3^ s, the strain output at different temperatures was significantly different. The change in strain output at room temperature was faster than in the temperature range of 200 °C–1000 °C. Between 6.0 × 10^−4^ and 1.6 × 10^−3^ s, the corresponding strain outputs of 200 °C and 600 °C were lower than at 800 °C and 1000 °C. The strain values at different temperatures tended to be gradually stable over time. The difference between the corresponding stable values at different temperatures was ±0.05 × 10^−5^.

### 2.5. Equal-Strength Beam Finite Element Analysis of the GFs of PtRh6 TFSGs

The GFs of the PtRh6 TFSGs at different temperatures were obtained using the finite element method and mathematical modeling. The force per unit area on the surface of an equal-strength beam generates strain and transmits it to the surface of the strain gauge, which deforms it. By connecting Equation (4) with the following mathematical model, the changes in resistivity and strain sensitivity coefficient of the strain gauge at different temperatures can be obtained. The mathematical model was as follows:

(5) $$\frac{{\Delta l}_{x}}{l_{x0}}=\frac{\int_{\varepsilon_{x0}}^{\varepsilon_{x1}}\varepsilon_{x}l_{x}{d\varepsilon }_{x}+{\left\{\int_{T_{0}}^{T}\left[Q\left(T\right)\right]\mathrm{dT}\right\}}_{x}-l_{x0}}{l_{x0}}$$

(6) $$\frac{\Delta S}{S_{0}}=\frac{\int_{\varepsilon_{y0}}^{\varepsilon_{y1}}\int_{\varepsilon_{z0}}^{\varepsilon_{z1}}\varepsilon_{y}l_{y}\cdot \varepsilon_{z}l_{z}{d\varepsilon }_{z}{d\varepsilon }_{y}+{\left\{\int_{T_{0}}^{T}\left[Q\left(T\right)\right]\mathrm{dT}\right\}}_{y}\cdot {\left\{\int_{T_{0}}^{T}\left[Q\left(T\right)\right]\mathrm{dT}\right\}}_{z}-S_{0}}{l_{x0}}$$

(7) $$\rho =\rho_{0}+\alpha \left(T-T_{0}\right)$$

(8) $$\mathrm{GF}=\frac{\Delta R}{\varepsilon \cdot R}$$

where $\varepsilon_{x}$, $\varepsilon_{y}$, and $\varepsilon_{z}$ are the strain variables along the equal-strength beam in the directions of x, y, and z, respectively; $l_{x0}$ is the initial length of the sensitive grid; $l_{y}$ and $l_{z}$ are the lengths of the sensitive grid after $\varepsilon_{y1}$ and $\varepsilon_{z1}$ are generated in the directions of y and z, respectively; and $S_{0}$ is the cross-sectional area of the sensitive grid when the equal-strength beam is unstressed. Since the self-weight of the equal-strength beam was neglected, $\varepsilon_{x0}$ = $\varepsilon_{y0}$ = $\varepsilon_{z0}$ = 0. $\rho_{0}$ = 1.669 × 10^−4^ Ωmm is the initial resistivity, α = 9.74 × 10^−9^ Ω·mm/°C is the resistivity temperature coefficient, $T_{0}$ is the room temperature, and T is the operating temperature, R is the initial resistance, ΔR is the change in resistance, and ε is the simulated strain output of the strain gauge.

**Figure 7:**
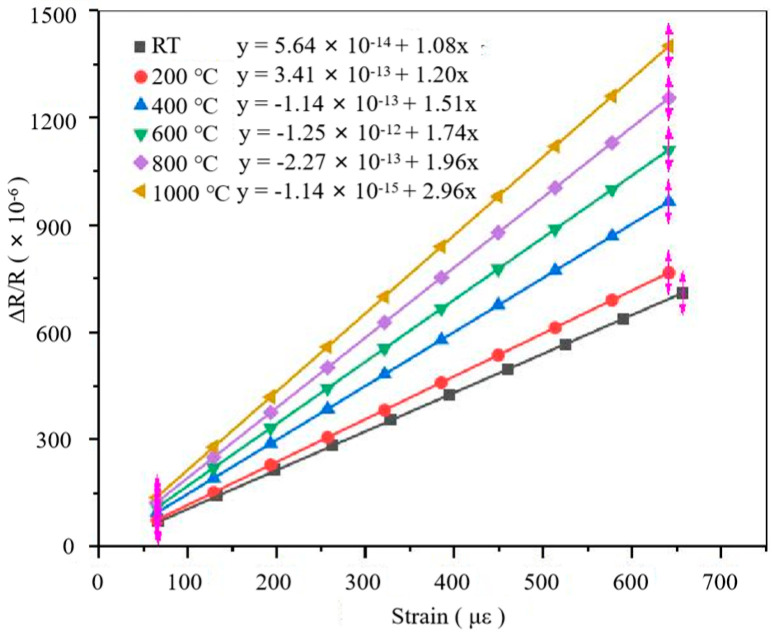
ΔR*/*R vs. ε relationship diagram of PtRh6 TFSG at different temperatures.

As shown in Figure 7, the ΔR*/*R vs. ε curves of the PtRh6 TFSGs at different temperatures were fitted, and their GF was 1.08 at room temperature and 2.19 at 1000 °C. The main reason the GF increased with the increase in temperature was that the resistivity increased with the increase in temperature, increasing the resistance change.

### 2.6. Equal-Strength Beam PtRh6 High-Temperature TFSGs TCR Finite Element Analysis

**Figure 8:**
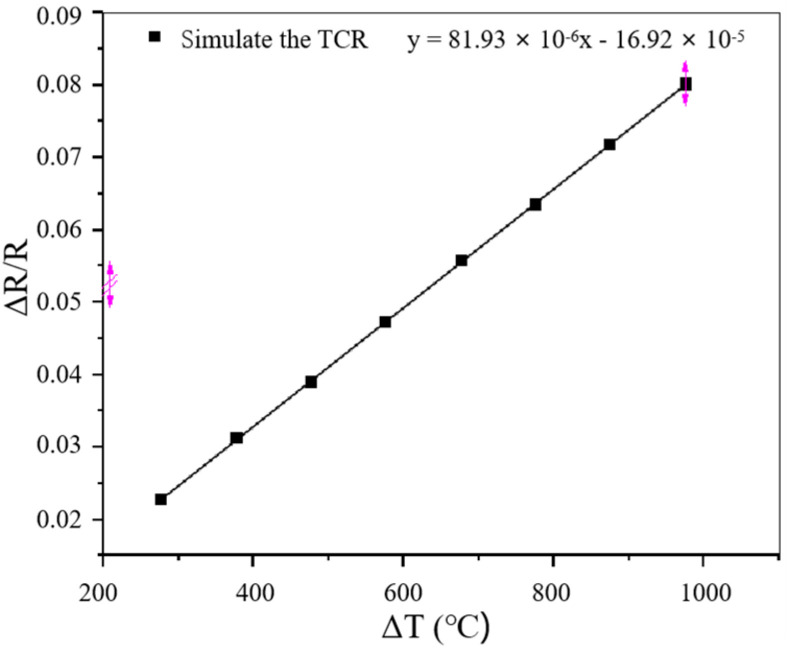
ΔR/R vs. ΔT relationship line diagram.

The TCR is another critical parameter of a TFSG and directly affects the accuracy of the output result. According to the simulation data in Section 2.5, the simulated TCR of the PtRh6 high-temperature TFSG was fitted. The results are shown in Figure 8. The TCR of the TFSG was 81.93 ppm/°C. The expression for the TCR is

(9) $$\mathrm{TCR}=\frac{R_{T}-R_{0}}{R_{0}\left(T-T_{0}\right)}$$

where $R_{T}$ is the resistance at temperature T and $R_{0}$ is the initial resistance at temperature $T_{0}$. Combining Equations (4) and (7) gives

(10) $$\mathrm{TCR}=\frac{\int_{T_{0}}^{T}\rho_{0}+\alpha \mathrm{TdT}\frac{L}{S}-R_{0}}{R_{0}\left(T-T_{0}\right)}$$

## 3. Experiment

### 3.1. Sample Preparation Process

**Figure 9:**
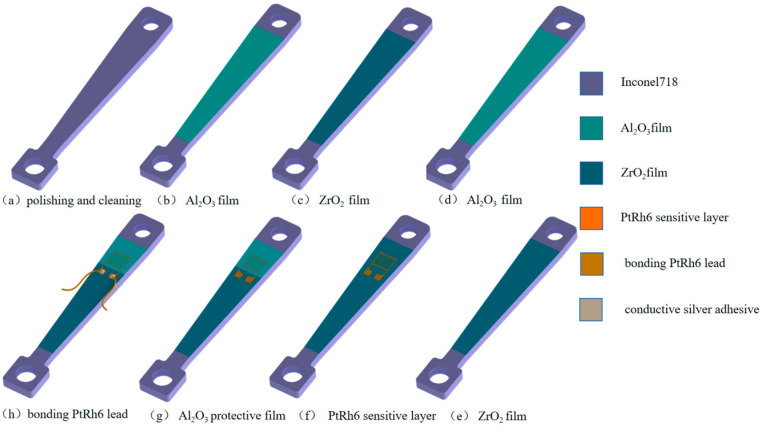
Inconel718 high-temperature nickel-based alloy equal-strength beam PtRh6 TFSG preparation flow chart.

A PtRh6 high-temperature TFSG was prepared on the surface of an Inconel 718 high-temperature nickel-based alloy. The preparation process is shown in Figure 9. The substrate surface was polished with 200 grit, 400 grit, 600 grit, and 800 grit sandpaper and measured using a TR150A surface roughness test and was found to have a surface roughness of 0.025 μm. The sample was placed in an ultra-high-sonic cleaning oscillator with acetone, ethanol, and deionized water for 20 min [32]. We wiped the surface with an absorbent paper towel and dried it with nitrogen. Al_2_O_3_/ZrO_2_/Al_2_O_3_/ZrO_2_ composite insulating layers were prepared on the surface of the substrate. In order to ensure a sufficient bonding force between the composite insulating layers, the composite insulating layer was heat-treated. The temperature was maintained at 600 °C for 1 h. The PtRh6 sensitive layer was prepared using the mechanical mask method and a DC pulse magnetron sputtering coater. Because of the smaller size of the sensitive grating and the lower temperature of the sputtering vacuum chamber, the TFSG quickly fell off. Therefore, an annealing treatment at 900 °C for 3 h using the mechanical mask method covered the electrode of the sensitive layer and deposited an Al_2_O_3_ protective layer of 800 nm in a DC pulsed magnetron sputtering coater. Finally, the lead wires were connected to the substrate with high-temperature conductive silver glue. The highest heat-resistant temperature of the high-temperature conductive silver glue was 1301 °C, the lead wire was PtRh6, and the lead wire resistance was about 1.45 Ω.

### 3.2. Insulation Film Preparation

The Al target and Zr target with 99% purity were selected to prepare single-layer Al_2_O_3_ and ZrO_2_ films, a double-layer Al_2_O_3_/ZrO_2_ composite insulating film with a film thickness ratio of 3:2, and a four-layer composite insulating film Al_2_O_3_/ZrO_2_/Al_2_O_3_/ZrO_2_ with a film thickness ratio of 11:4:11:4, with a total thickness of approximately 1.5 μm. The preparation was carried out using the DC pulse magnetron sputtering technique. The process parameters are shown in Table 6.

**Figure 10:**
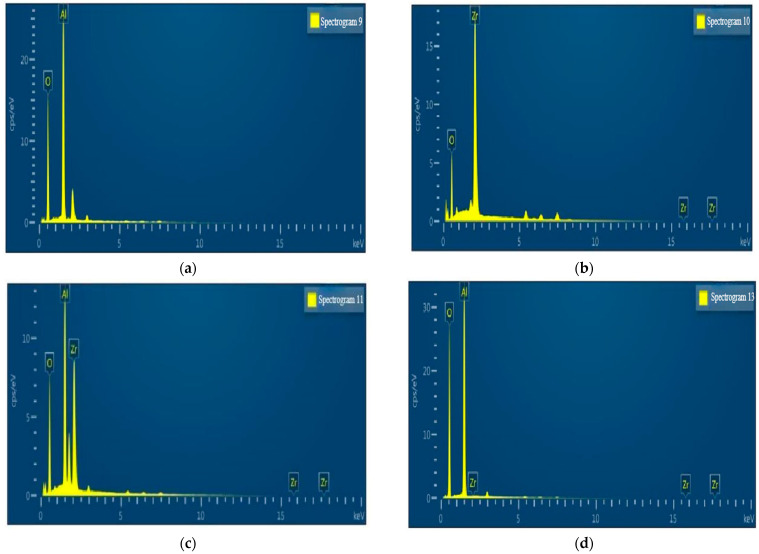
Component content analysis of the four insulation layers. (**a**) Single-layer Al_2_O_3_ film, (**b**) Single-layer ZrO_2_ film, (**c**) Double-layer Al_2_O_3_/ZrO_2_ film and (**d**) Four-layer Al_2_O_3_/ZrO_2_/Al_2_O_3_/ZrO_2_ film.

The EDS composition analysis results of the four insulating layers are shown in Figure 10. The single-layer ZrO_2_ and Al_2_O_3_/ZrO_2_ double-layer composite structure insulating layers contained relatively low amounts of oxygen. In contrast, the Al_2_O_3_/ZrO_2_/Al_2_O_3_/ZrO_2_ composite structure insulating layers contained higher amounts of oxygen. This showed that the structure could lock in high-valence positive charges at high temperatures, improving the overall ability to lock in oxygen at high temperatures, augmenting the ZrO_2_ high-temperature conductivity with this deficiency, and improving the overall high-temperature insulation stability.

**Figure 11:**
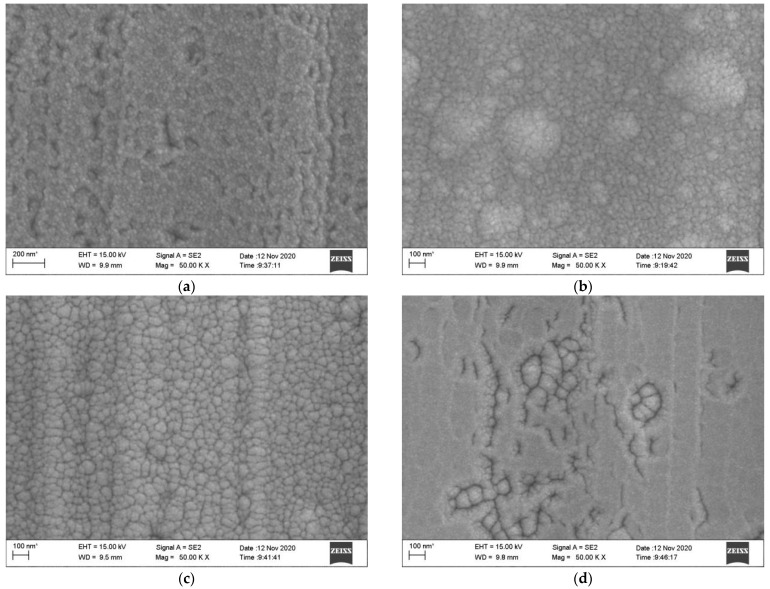
SEM images of insulating layers with different structures. (**a**) Single-layer Al_2_O_3_ film, (**b**) Single-layer ZrO_2_ film, (**c**) Double-layer Al_2_O_3_/ZrO_2_ film and (**d**) Four-layer Al_2_O_3_/ZrO_2_/Al_2_O_3_/ZrO_2_ film.

As shown in Figure 11, scanning electron microscopy (SEM) was used to observe the microstructure of different insulating layers. This showed that the particle surfaces of the single-layer and double-layer composite films had no significant gaps and holes, and the surfaces were compact but uneven. A few particle mounds appeared on the surface of the monolayer ZrO_2_, streak-like bumps appeared on the surface of the bilayer composite structure, and some cracks existed on the surface of the four-layer composite structure. However, the rest of the surface was flat and compact.

### 3.3. High-Temperature Insulation Test

**Figure 12:**
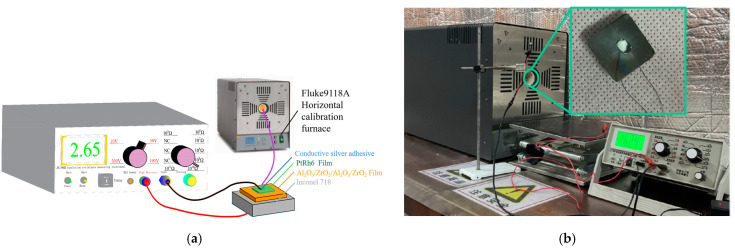
High-temperature insulation resistance test: (**a**) simulation diagram, and (**b**) field experiment map.

After comparing the high-temperature stability of Al_2_O_3_ and ZrO_2_, it was determined that the two kinds of composite insulation layers were Al_2_O_3_ films in contact with a high-temperature nickel-base alloy matrix. After the preparation of the insulating film, PtRh6 electrodes were prepared on the surface of the insulating layer and the opposite substrate surface. A platinum–rhodium wire with a diameter of 0.1 mm was used as the lead wire. In order to ensure excellent contact between the PtRh6 lead wire and the electrode, the lead wire was flattened, fixed with the substrate, coated with conductive silver glue, and placed in a Fluke9118A horizontal temperature-controlled furnace at room temperature for 24 h. The high-temperature insulation resistance test results are shown in Figure 12. We set the experimental temperature range to 300 °C–1000 °C, the temperature increase rate to 10 °C/min, maintained the temperature for 10 min every 100 °C increase, and recorded the insulation resistance value at each temperature point.

**Figure 13:**
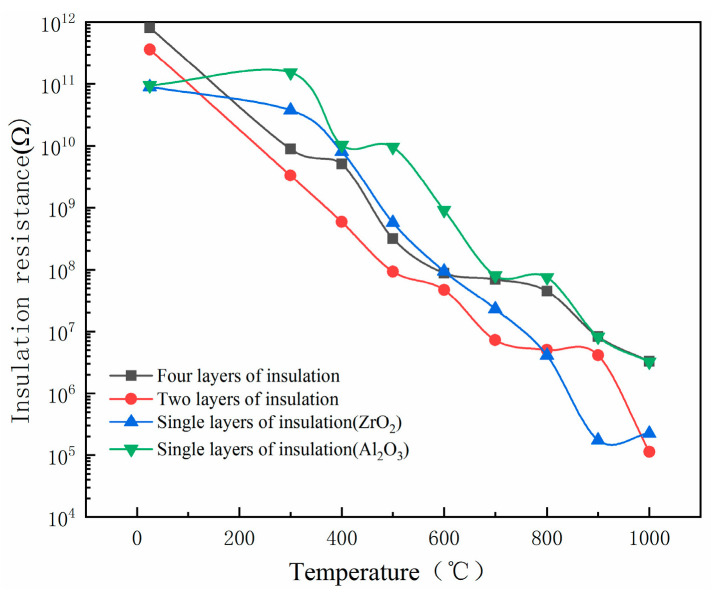
Comparison of the insulation resistance of different structural insulation layers.

As shown in Figure 13, the insulation resistance decreased with the increase in temperature. However, when the temperature reached 1000 °C, only single-layer Al_2_O_3_ and four-layer insulation films met the insulation requirements. At room temperature, the four-layer insulation film had a maximum resistance of 8.14 × 10^5^ MΩ, and the highest insulation resistances at 1000 °C were found for the single-layer alumina and four-layer insulation films. Although the insulation resistance was similar at high temperatures, the insulation of the four-layer insulation film was higher at room temperature.

### 3.4. Effect of the Annealing Temperature on the Insulation Properties of the Four-Layer Composite Insulating Film

During the film preparation process, internal stress is generated in and between films, and natural preservation can lead to the fracture and peeling of the insulating film. Therefore, it is necessary to heat treat the Al_2_O_3_/ZrO_2_/Al_2_O_3_/ZrO_2_ composite insulating film. In this study, the Al_2_O_3_/ZrO_2_/Al_2_O_3_/ZrO_2_ composite insulation layer prepared using the DC pulse magnetron sputtering technique was subjected to an annealing heat treatment in a muffle furnace.

**Figure 14:**
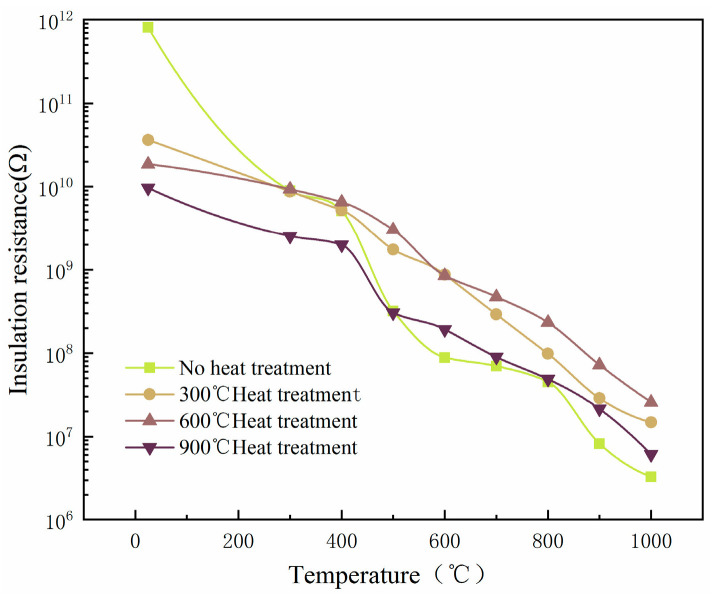
Effect of different annealing temperatures on the insulation resistance.

The prepared composite insulating film specimens were placed in a box muffle furnace for the high-temperature annealing treatment and annealed at 300 °C, 600 °C, and 900 °C for 3 h with a heating rate of 5 °C/min and kept for 1 h. The effects of different annealing temperatures on the insulation were compared. As shown in Figure 14, the resistance of the unannealed composite insulating film changed more obviously with temperature. The annealed composite insulating film had poor initial insulation but was less affected by temperature. After annealing at 300 °C, the insulation resistance of the composite insulating film stayed at around 10^10^ Ω when working from 25 °C to 400 °C, but when the working temperature exceeded 400 °C, the insulation resistance decreased; after annealing at 900 °C, the composite insulating film had a substantial fluctuation in insulation resistance only between 400 °C and 500 °C. When the working temperature was more than 500 °C, the insulation resistance at different annealing temperatures gradually appeared. Therefore, the composite insulation film after 3 h of annealing heat treatment at 600 °C had the best insulation, and the insulation resistance reached 25.8 MΩ when the temperature reached 1000 °C.

### 3.5. Calibration of the Gauge Factor

**Figure 15:**
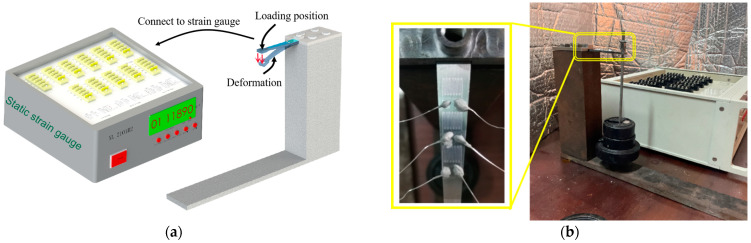
Static calibration experimental setup: (**a**) simulation diagram and (**b**) field experiment map.

**Figure 16:**
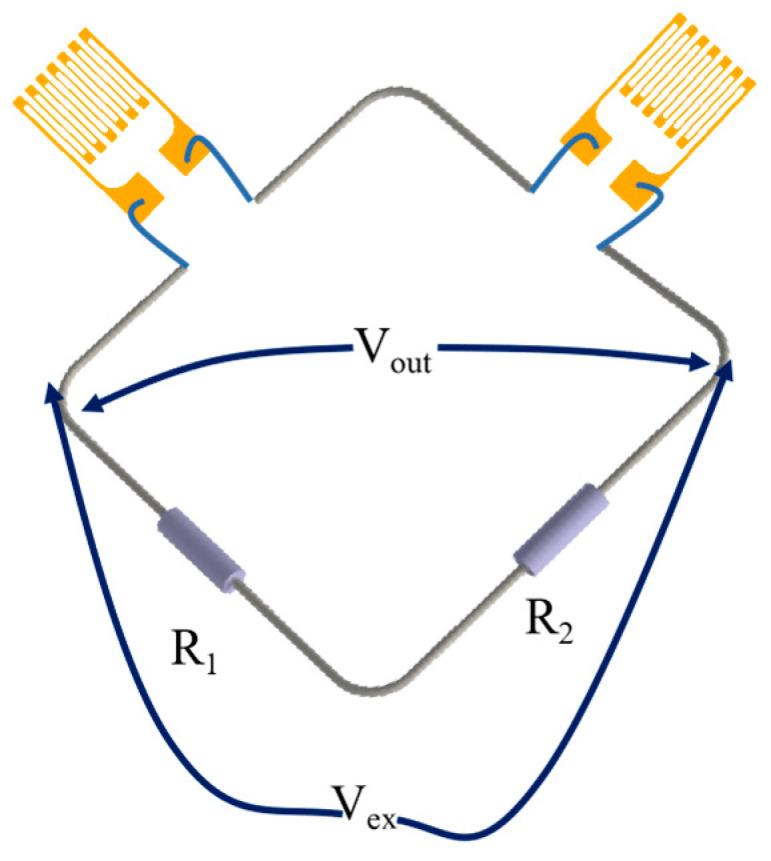
Schematic diagram of the strain transducer connected to the Wheatstone half-bridge circuit.

**Figure 17:**
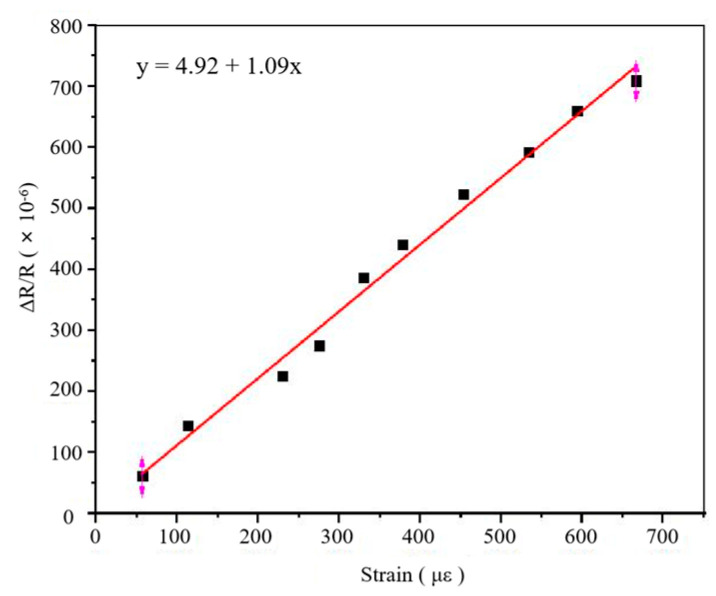
Calibration of the strain sensitivity coefficient.

The static calibration test settings are shown in Figure 15. The free end of the equal beam was loaded to 50 kg in steps of 5 kg, and the loading test was repeated three times; the average value of three results was obtained. In order to improve the accuracy of the strain output, a Wheatstone half-bridge circuit was lapped, as shown in Figure 16, and a static strain gauge XL 2101B2 was used to measure the strain, which was combined with Equation (8) to obtain the sensitivity coefficient of the PtRh6 high-temperature TFSG. As seen in Figure 17, there was an excellent linear relationship between the resistance change rate and strain. The sensitivity coefficient of the PtRh6 high-temperature thin-film strain gauge was 1.09, which was close to the simulated sensitivity coefficient of 1.08.

### 3.6. Calibration of TCR before and after Heat Treatment

**Figure 18:**
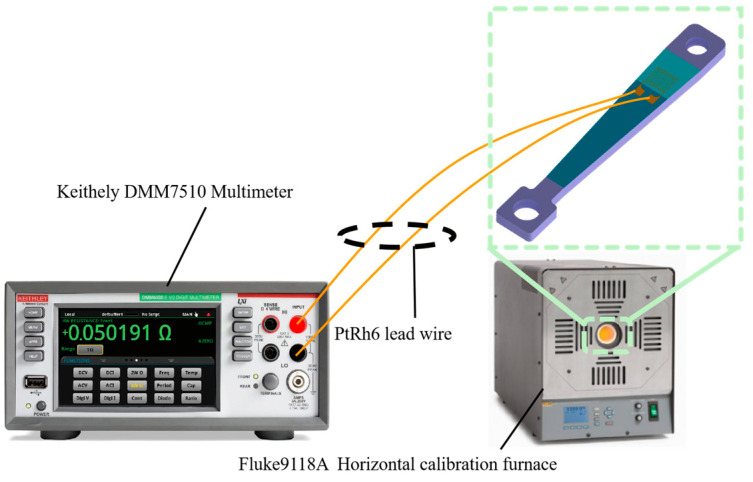
Construction of the test bench for the temperature TCR.

**Figure 19:**
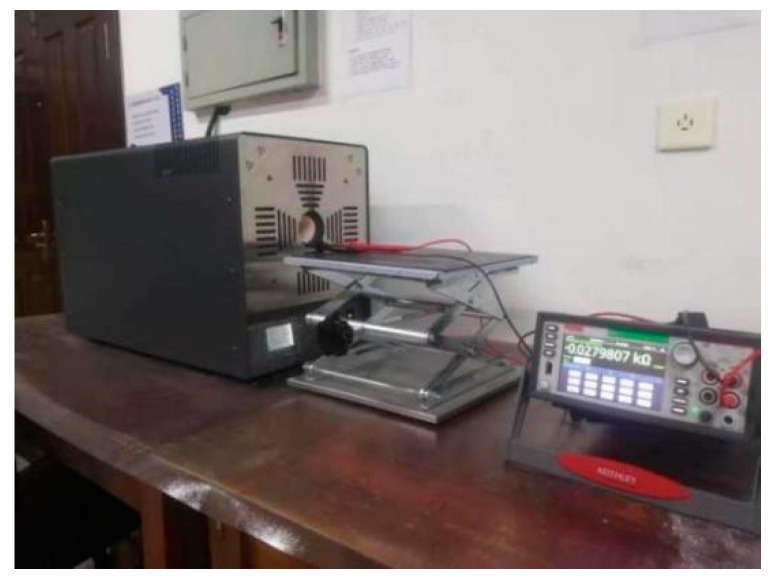
TCR test field experiment diagram.

The equal-strength beam PtRh6 high-temperature TFSG samples were placed in a muffle furnace, set at 900 °C, and annealed for 3 h. The sample was placed in a Fluke 9118A high-temperature metering furnace before and after annealing. The temperature of the metering furnace was adjusted to achieve gradual heating of the thin-film strain gauge, which was connected to a Keithley DMM7510 multimeter to record the resistance value of the sensitive layers at different temperatures and the TCR coefficient calculated using Equation (9). The construction of the calibration resistance temperature coefficient experimental bench is shown in Figure 18 and Figure 19.

**Figure 20:**
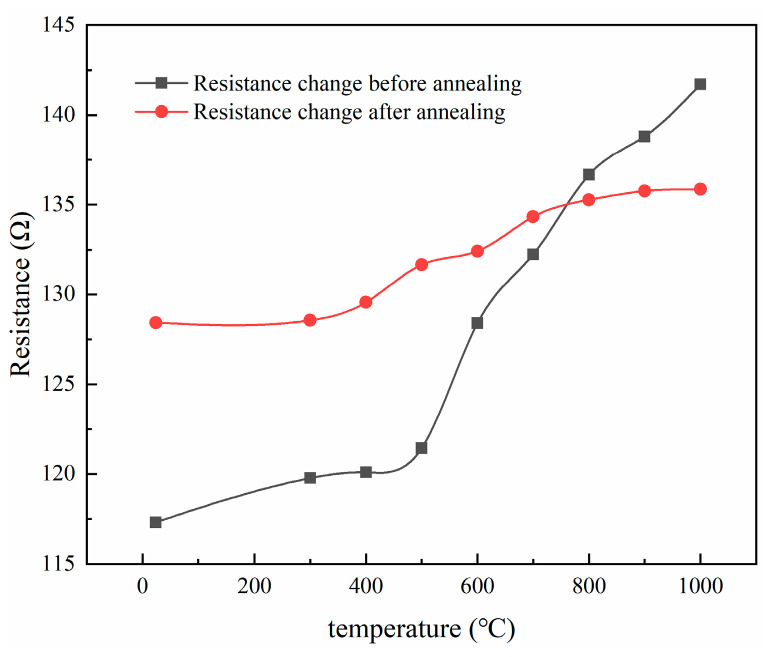
The resistance change of the strain gauge before and after annealing.

**Figure 21:**
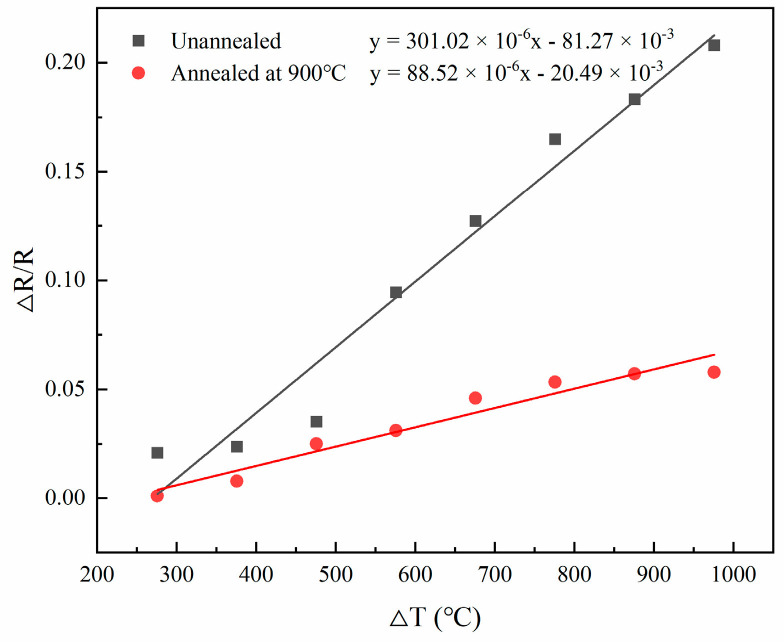
TCR of the strain gauge before and after annealing.

Figure 20 shows that in equal-strength beam PtRh6 high-temperature TFSGs without annealing, the amount of resistance change from room temperature to 500 °C was 4.116 Ω, and from 500 °C to 1000 °C, it was 20.274 Ω, indicating oxidation of the unannealed thin-film strain gauges. The film structure was stable at temperatures below 500 °C; at temperatures above 500 °C, the degree of oxidation was greater. The total change in resistance from room temperature to 1000 °C after annealing the TFSG was 7.434 Ω, indicating that the annealing heat treatment improved the film’s stability. Because of the oxidation that occurred, the initial resistance of the film after the heat treatment was more significant than the initial resistance before annealing, and the amount of resistance change became significantly smaller. According to Figure 21, the TCR values before and after annealing were 301.02 ppm/°C and 88.52 ppm/°C, respectively, which were found using a linear fit, and the TCR after annealing was similar to the simulated TCR result of 81.93 ppm/°C.

**Figure 22:**
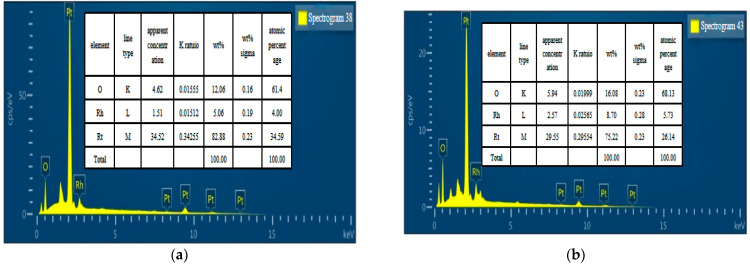
EDS composition analysis: (**a**) before annealing and (**b**) after annealing.

In order to accurately analyze the relationship between the resistance temperature coefficient and the heat treatment, a compositional analysis (EDS) was carried out on the PtRh6 films before and after annealing. As can be seen from Figure 22, the percentage of elemental oxygen was 61.4% before annealing and increased to 63.18% after annealing, indicating that slight oxidation of the PtRh6 sensitive layer occurred; thus, the initial resistance of the sample after the atmospheric annealing heat treatment was high.

**Figure 23:**
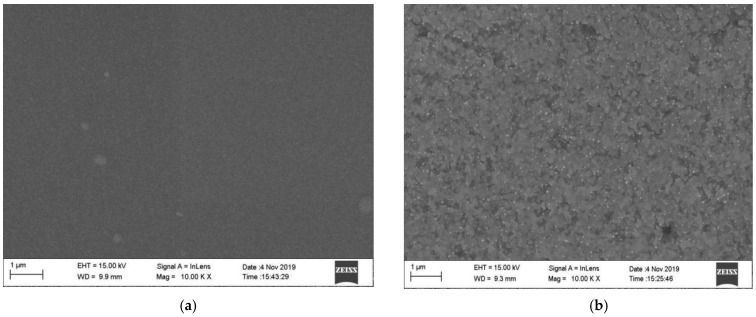
SEM comparison of PtRh6 films before and after annealing: (**a**) before annealing and (**b**) after annealing at 900 °C.

Figure 23a depicts the unheated film, which was uniformly dense except for a few mounds of particles on the surface. Further heat treatment of the PtRh6 film was required to obtain a high-quality film. Figure 23b depicts the PtRh6 film after the heat treatment, in which white crystals appeared on the surface, the surface lattice became more extensive, and the internal stress was ultimately released. Heat treatment made the lattice bigger and it reached equilibrium; the larger structure increased the binding force between particles and the whole structure was compact. Therefore, the above analysis showed that annealing reduced the temperature coefficient of resistance of the PtRh6 strain gauges and improved the performance of the strain gauges in high-temperature environments.

In this study, a PtRh6 high-temperature TFSG with integrated functional thin films was deposited on an Inconel718 high-temperature nickel-based alloy material using magnetron sputtering technology, and the Al_2_O_3_/ZrO_2_/Al_2_O_3_/ZrO_2_ four-layer insulating film composite structure was explored and annealed. During treatment, the insulation resistance of the four-layer film at 1000 °C was as high as 25.8 MΩ, which showed excellent insulation performance. The temperature coefficient of resistance of the strain gauge was measured to be 88.52 ppm/°C, and the strain sensitivity coefficient was 1.09. Thus, we produced a new functional material for measuring the strain of aero-engine turbine blades in a high-temperature environment above 600 °C. Table 8 summarizes the TFSG prepared from typical high-temperature materials. By comparing the deposition methods of different films, substrate materials, maximum operating temperature, GF, and TCR, it can be seen that for metals and alloys, the GF was 1.0–4.0. The GF of the ceramic material TiB_2_/SiCN was as high as 7.12, but its TCR could not be given, and the operating temperature was only 700 °C. The working temperature of the PtRh6 film was as high as 1000 °C. Future research may focus on its performance and improve its GF and TCR. PtRh6 may thus be an excellent material to use for strain gauges.

## 4. Conclusions

In this study, the high-temperature stability of Inconel718 high-temperature nickel-based alloy equal-strength beam PtRh6 TFSGs was studied by combining finite element analysis and experimental procedures, and the following conclusions were drawn:

(1) By establishing an Inconel718 high-temperature nickel-based alloy equal-strength beam PtRh6 TFSG thermo-mechanical coupling finite element model, the variation law of the strain output with time at different temperatures was studied. It was found that before 1.6 × 10^−3^ s, the strain output at different temperatures was different. The strain output at room temperature changed faster than that in the temperature range of 200 °C to 1000 °C. However, the strain values at different temperatures gradually became stable with time, and the difference between the stable values at different temperatures was ±0.05 × 10^−5^.

(2) A single-layer Al_2_O_3_ insulating film, single-layer ZrO_2_ insulating film, double-layer Al_2_O_3_/ZrO_2_ composite insulating film, and four-layer Al_2_O_3_/ZrO_2_/Al_2_O_3_/ZrO_2_ composite insulating film were prepared on an Inconel718 high-temperature nickel-based alloy equal-strength beam. Using a Fluke 9118A to provide a high-temperature environment, the insulation properties of the above four insulating film structures at high temperatures were tested. The results showed that the insulation resistances at 1000 °C were 0.224 MΩ, 3.23 MΩ, 0.113 MΩ, and 3.296 MΩ, respectively. Therefore, the four-layer composite insulating film was selected as the insulating layer of the PtRh6 high-temperature TFSG. Furthermore, by annealing the four-layer insulating film at 600 °C for 3 h and 1 h of heat preservation, the insulation resistance was as high as 25.8 MΩ.

(3) By analyzing the stress transfer hysteresis of the Inconel718 high-temperature nickel-based alloy equal-strength beam PtRh6 TFSGs, it was determined that the optimal sensitive layer film thickness and sensitive grid length of the PtRh6 TFSG were 800 nm and 4 mm, respectively. After annealing the prepared Inconel718 high-temperature nickel-based alloy equal-strength beam PtRh6 TFSGs at 900 °C for 3 h, the TCR and GF were experimentally calibrated. The TCR was 88.52 ppm/°C and the GF at room temperature was 1.09, while the TCR obtained by the finite element simulation was 81.93 ppm/°C and the GF at room temperature was 1.08. This verified the reliability of the Inconel718 high-temperature nickel-based alloy equal-strength beam PtRh6 TFSG finite element simulation model.

In future research, we will focus on solving the problems that arise in sensors when they work in a harsh environment. Several bending cycles (>1000) will be carried out to study the resistance of the insulating film and the influence of the GF and TCR of the strain gauge to improve the performance of the PtRh6 thin film.

## Figures and Tables

**Table 1:** Dimensions of the longitudinal grid areas for different thicknesses of sensitive layers.

Film Thickness	Longitudinal Length	Longitudinal Width	Crossbar Length	Crossbar Width	Electrode Length	Electrode Width
600 nm	3 mm	0.1 mm	0.6 mm	0.5 mm	2 mm	2 mm
800 nm	4 mm	0.1 mm	0.6 mm	0.5 mm	2 mm	2 mm
1000 nm	5 mm	0.1 mm	0.6 mm	0.5 mm	2 mm	2 mm
1200 nm	6 mm	0.1 mm	0.6 mm	0.5 mm	2 mm	2 mm

**Table 2:** Physical parameters of the layers of the thin-film sensor material.

Functional Layer	Materials	Young’s Modulus (GPa)	Poisson’s Ratio	Shear Modulus (GPa)
Protective layer	Al_2_O_3_	152	0.2	83.33
Sensitive layer	PtRh6	169	0.38	61
Insulation layer	Al_2_O_3_	152	0.2	83.33
	ZrO_2_	220	0.3	119
Alloy substrates	Inconel718	227.79	0.3241	137

**Table 3:** Steady-state strain output values for different film thicknesses at different temperatures.

Film Thickness Temperature	200 °C Loading	200 °C Unloading	600 °C Loading	600 °C Unloading	1000 °C Loading	1000 °C Unloading
Substrate surface strain	3.314 × 10^−4^	1.477 × 10^−7^	3.298 × 10^−4^	1.477 × 10^−7^	3.305 × 10^−4^	1.477 × 10^−7^
600 nm	3.218 × 10^−4^	1.474 × 10^−7^	3.222 × 10^−4^	1.525 × 10^−7^	3.222 × 10^−4^	1.794 × 10^−7^
800 nm	3.231 × 10^−4^	1.479 × 10^−7^	3.232 × 10^−4^	1.532 × 10^−7^	3.232 × 10^−4^	1.477 × 10^−7^
1000 nm	3.202 × 10^−4^	1.473 × 10^−7^	3.205 × 10^−4^	1.531 × 10^−7^	3.205 × 10^−4^	1.794 × 10^−7^
1200 nm	3.199 × 10^−4^	1.474 × 10^−7^	3.199 × 10^−4^	1.531 × 10^−7^	3.199 × 10^−4^	1.795 × 10^−7^

**Table 4:** Geometric dimensions and resistance parameters of the functional areas of the sensitive layers of the TFSG.

Functional Area	Length (mm)	Width (mm)	Film Thickness (nm)	Resistance (Ω)	Quantity	Total Resistance (Ω)
Longitudinal bar	4	0.1	800	8.3	12	91.3
Crossbar	0.6	0.5	800	5.81	11	11.62
connecting longitudinal bar	6.6	0.2	800	0.249	2	2.49
Connecting the Crossbar	0.6	0.6	800	0.415	3	0.83
Electrode area	2	2	800	0.415	2	0.83

**Table 5:** Inconel718 and PtRh6 linear coefficients of thermal expansion (α/10^−6^ K^−1^).

Temperature (°C) Materials	100	200	300	400	500	600	700	800	900	1000
Inconel718	14.7	14.7	14.8	14.8	14.9	15.2	15.7	15.8	15.9	15.9
PtRh6	9.0	9.0	9.0	9.0	9.015	9.17	9.22	9.23	9.23	9.23

**Table 6:** Insulation film preparation process parameters.

Materials	Target Materials	Ar:O (sccm)	Sputtering Air Pressure (Pa)	Background Vacuum (Pa)	Sputtering Power (W)
Al_2_O_3_	Al	20:8	0.6	1 × 10^−3^	400
ZrO_2_	Zr	20:8	0.6	1 × 10^−3^	400

**Table 8:** Summary of the operating temperatures of different film materials.

Sensitive Layer Materials	Substrate	Fabrication Method	Maximum Use Temperature (°C)	Gauge Factor	TCR (ppm/°C)	Reference
Ni80Cr20	DIN 50,125 Form H	Sputtering	200	2.05	−51.5	[7,8]
PdCr	K456	Sputtering	800	1.78–2.13		[9]
TiAlN	sapphire	Sputtering	350	2.5		[11]
Pt	96% Al_2_O_3_ ceramic	Sputtering	850	1.9–2.5		[12]
TiB_2_/SiCN	Ni-based superalloy	DIW	700	7.12		[33]
PtRh6	Inconel718	Sputtering	1000	1.09	88.52	This work

## Data Availability

Not applicable.
